# Assessment of inhibitory potential of essential oils on natural mycoflora and *Fusarium* mycotoxins production in wheat

**DOI:** 10.1186/1752-153X-7-32

**Published:** 2013-02-14

**Authors:** Renata-Maria Sumalan, Ersilia Alexa, Mariana-Atena Poiana

**Affiliations:** 1 Banat’s University of Agricultural Sciences and Veterinary Medicine from Timisoara, Faculty of Horticulture and Forestry, Calea Aradului 119, Timisoara, RO 300645, Romania; 2 Banat’s University of Agricultural Sciences and Veterinary Medicine from Timisoara, Faculty of Food Processing Technology, Calea Aradului 119, Timisoara RO 300645, Romania

**Keywords:** Essential oils, Wheat, Antifungal activity, *Fusarium* mycotoxins, Antioxidant properties

## Abstract

**Background:**

In the last years essential oils from different plants were used in the prevention of fungi and mycotoxins accumulation in cereals. The most attractive aspect derived from using of essential oils as seed grains protectants is due to their non-toxicity. This study was focused on assessment the inhibitory effect of some essential oils: *Melissa officinalis* (O1), *Salvia officinalis* (O2), *Coriandrum sativum* (O3), *Thymus vulgaris* (O4) *Mentha piperita* (O5) and *Cinnamomum zeylanicum* (O6) against natural mycoflora and *Fusarium* mycotoxins production correlated with their antioxidants properties.

**Results:**

All essential oils showed inhibitory effect on fungal contamination of wheat seeds. This ability was dose-dependent. The highest inhibitory effect on *Fusarium* and *Aspergillus* fungi was recorded after 5 days of treatment. Fungi such as yeast (*Pichia, Saccharomyces and Hyphopichia*) were predominantly on seeds mycoflora after 22 days. Each treatment had a selective inhibitory effect on frequency of fungus genera. After 5 days of treatment the most fungicidal effect was recorder for O4, followed by O1. In terms of essential oils effect on mycotoxins development, the best control on fumonisins (FUMO) production was recorded for O6. The antioxidant properties of essential oils decreased in order: O4 > O1 > O6 > O5 > O2 > O3. Also, our data suggested that there is a significant negative correlation between antioxidant properties and seed contamination index (SCI), but there was not recorded a good correlation between antioxidant properties and FUMO content.

**Conclusions:**

Based on proven antifungal and antimycotoxin effects as well as their antioxidant properties, the essential oils could be recommended as natural preservatives for stored cereals. The highest inhibition of fungal growth was noted after 5 days of treatment and decreased after 22 days.

## Introduction

Quality assurance and safety of cereals has determined identifying of new alternative ways to preserve the nutritional value of grains. Using of medicinal plants from spontaneous flora in medical and nutritional purposes is practiced from ancient times. In micro-ecological favourable conditions, both on the field before harvest, and especially during storage for longer periods of time in inadequate conditions grains are exposed to fungal contamination, being favourable medium for moulds development. Among them, representatives of the genera *Alternaria, Cladosporium, Fusarium, Aspergillus* and *Penicillium* are known to have negative impact on the preservation of grains determining quantitative and qualitative losses [1].

*Fusarium* moulds have become a serious problem because they produce a range of toxic metabolites (mycotoxins) which endanger the health of both humans and animals. Although *Fusarium* species are predominantly considered as field fungi, it has been reported that FUMO production can occur post-harvest when storage conditions are inadequate [2]. *Fusarium* species, *F. proliferatum* and *F. verticillioides* are the most prevalent species in freshly harvested corn [3-6]. Fumonisins (FUMO) and deoxynivalenol (DON) are two important *Fusarium* mycotoxins that have received considerable attention related to food safety. A selected number of *F. proliferatum* isolates showed FUMO production capability on autoclaved rice seeds [7]. Recent researches have indicated the presence of three forms of FUMO produced by *Fusarium* species during the post-harvest wheat [8].

The prevention is the best method for controlling fungi and mycotoxins contamination. Post harvest treatment with antifungal agents has been examined to ensure that control can be achieved [3]. Previously studies tested the effect of food grade antioxidants such as propyl paraben (PP), butylated hydroxyanisole (BHA) and butylated hydroxytoluen (BHT) to control *Fusarium* species and mycotoxins production [4,5].

Nowadays is emphasized the need to prevent fungal spoilage and mycotoxins accumulation by using of natural substances with fungicidal effects. In other recent study conducted by us it was assessed the potential of natural antioxidants derived from grape seed and pomace as by-products resulted in wine industry [9].

In the last years essential oils from different plants were used in the prevention of fungi and mycotoxins accumulation in cereals [10-12]. Essential oils, also known as volatile oils, are complex mixtures of volatile constituents biosynthesized by plants, which mainly include terpenes, terpenoids, aromatic and aliphatic constituents, all characterized by low molecular weight [13]. They are a valuable natural source of antioxidants and biologically active compounds. Due their bioactivity in the vapors phase, essential oils could be used as a fumigant for stored cereals protection [14]. The most attractive aspect derived from using of essential oils and/or their constituents as crop protectants is due to their non-toxicity [15].

The inhibitory effect of medicinal plants on growth rate of *Fusarium* species has been highlighted [14,16-19]. The results reported by Bluma *et al.*[20] have found that antifungal activity was strongly associated with monoterpenic phenols, especially thymol, carvacrol and eugenol, in the oils.

Many previous studies have been carried using essential oils in microbiological media, but only few studies were done in vivo to assess the antifungal effect of essential oils on opportunistic fungi of cereal seeds [21]. From these reasons, we have proposed to evaluate the antifungal and fungicidal effect of essential oils in vivo. In this regard, our study is focused on the assessment of inhibitory potential of essential oils extracted from aromatic and spice plants (*Mentha piperita, Melissa officinalis, Salvia officinalis, Coriandrum sativum, Thymus vulgaris* and *Cinnamomum zeylanicum*) against natural mycoflora of wheat seeds and *Fusarium* mycotoxins production in close correlation with their antioxidant properties. Natural essential oils are expected to be more advantageous than the synthetic agents due to their biodegradability and low toxicity. Considering the previous research suggestions on the fact that the levels of essential oils necessary to inhibit microbial growth are higher in foods than in culture media due the interactions between phenolic compounds and the food matrix, in our study we used the essential oils in the range of 500 and 2000 ppm [22].

## Experimental research

### Wheat samples and the essential oils

In this study naturally contaminated wheat grain (cv. Lovrin 34) harvested in 2010 in western part of Romania was used. The main physico-chemical characteristics of wheat grain were: humidity (12.8%), protein (12.5%) and gluten index (22%). The wheat grain samples, natural contaminated with 0.689 ppm FUMO and 0.420 ppm DON were weighed (2000 g) and chemically sterilized so that opportunistic mycoflora to be inactivated. The sterilization has done into sterile flask with hypochlorite solution 1:10 (v/v) followed by washing with distilled water twice. Flasks were shaken and equilibrated for 48 h at 4°C.

Six essential oils were tested for their inhibitory potential on natural mycoflora and *Fusarium* mycotoxins production in wheat. These essential oils from lemon balm (*Melissa officinalis* L.), garden sage (*Salvia officinalis* L.), coriander (*Coriandrum sativum* L*.*), thyme (*Thymus vulgaris* L*.*), peppermint (*Mentha piperita* L.) and cinnamon (*Cinnamomum zeylanicum* L*.*) were obtained from SOLARIS PLANT SRL, Romania.

### Chemical reagent and microbiological medium

Commercial ELISA kits for mycotoxins identification were purchased from R-Biopharm: DON R5901, FUMO R5602. The ELISA method validation was carried on reference certificated materials produced by R - Biofarm.

For analysis of natural seeds mycobiota were used potato dextrose agar medium (PDA) - potatoes infusion 20%, dextrose 2% and agar 1.5%, with pH adjusted at 5.6 ± 0.2 and dichloran chloramphenicol peptone agar medium (DCPA) for identification of *Fusarium* species: peptone 1.5%, KH_2_PO_4_ 0.1%, MgSO_4_ 0.05%, chloramphenicol 0.01%, dichloran 0.2% in ethanol (w/v), agar 1.5%.

### Experimental samples

The wheat samples (100 g) were spiked separately with the six essential oils at three levels (500, 1000 and 2000 ppm). The treatment was performed in sterile Petri dishes (Ø=120 mm). The wheat seeds were disposed in a single layer covering the whole surface. All essential oils were diluted previously in ethanol 96% (v/v) so that 1 ml of alcoholic solution ensured a level of 500, 100 and 2000 ppm related to the wheat seeds weight. Also, the control samples were treated with 1 ml ethanol. Distilled water was added to obtain the required a_w_ (0.900). The amount of water was calculated based on moisture adsorption curve of the grains. In order to ethanol evaporation, the dishes were placed at 25°C for 2 hours and periodically mixed. Samples were incubated at 25°C for 22 days and a_w_ of wheat seeds samples were kept at 0.900 by weighing and spraying with sterile water. After 5 and 22 days the samples from each experiment were taken in order to fungi and mycotoxins assessment.

### The evaluation and identification of seeds mycobiota

The assessment of fungus was performed by direct plating method [23]. Ten subsamples of wheat seeds from each treatment were placed on PDA in Petri dishes (Ø=120 mm). The analysis of investigated parameters was performed initial as well as after 5 and 22 days of treatments with essential oils. The Petri dishes were incubated at 25 ± 2°C, in darkness and observations relating to fungal colonies growth on wheat seeds were visualized using stereo-binocular microscope. The number of contaminated seeds was used in order to estimate the seeds contamination index (SCI) according to Doolotkeldieva *et al.* using the formula (1) [24]:

(1) $$\text{SCI}\left(\%\right)=\frac{\text{number}\;\text{of}\;\text{contaminated}\;\text{seeds}}{\text{total}\;\text{number}\;\text{seeds}}\times 100$$

The identification of fungus genera has been performed according to Hocking *et al.*[25] and for *Fusarium sp.* in agreement with Leslie *et al.*[26].

In order to identify the species by presence or absence of microconidia, chlamidoconidia or chlamydospores, size and shape of macroconidia, the *Fusarium* genera were isolated and grown on DCPA medium. The frequency of occurrence of the fungal genera was calculated by formula (2) [24]:

(2) $$\text{Fr}\left(\%\right)=\frac{\text{number}\;\mathrm{of}\;\text{samples}\;\text{with}\;a\;\text{fungal}\;\text{genus}}{\text{total}\;\text{number}\;\mathrm{of}\;\text{samples}}\times 100$$

### Mycotoxins analysis

The method used in this study was enzyme-linked immunosorbent assay (ELISA). Sample preparation and analyzes were conducted according to the instructions outlined in the R-Biopharm kits ELISA. The ground samples (5 g) were extracted with 25 ml of methanol:water 70:30 (v/v) for FUMO analysis or using 100 ml distilled water for DON analysis and shaken in a warring blender at high speed for 20 min. The extract was filtered through a Whatman (Maidstone, UK) filter paper (No. 1). A 1-ml filtrate was diluted at 1:13 for FUMO. For DON the filtered extract was used directly for mycotoxins analysis. Standard solutions and prepared samples (50 μl) were mixed with 50 μl of enzyme conjugate in individual dilution wells. Antibody solution (50 μl) was added and mixed gently by shaking the plate manually and incubate for 10 min. at room temperature. The wells were washed three times with 250 μl distilled water. Substrate (100 μl) was added to each well and incubated for 5 min at room temperature. Stop solution (100 μl) was added to each well and the intensity of the resulting yellow colour was measured at a wavelength of 450 nm using ELISA 96-well plate reader (PR-1100, Bio-Rad Laboratories, USA). The mycotoxin losses were expressed as percentage related to the content registered in control sample.

### Total phenols assay

Total phenolic content of essential oils was determined using the Folin-Ciocalteu colorimetric method [27]. In order to extract total phenolic compounds from investigated essential oil samples, 1 g essential oil was mixed with 20 ml ethanol/water (70:30, v/v) by sonication at room temperature for 30 min. The mixture was centrifuged (5000 rpm, 10 min) and the supernatant was used for total phenolic analysis. A calibration curve using gallic acid was prepared and the absorbance of the standards and samples were measured at 750 nm using a UV–VIS spectrophotometer (Analytic Jena Specord 205). Results were expressed as μM gallic acid equivalents (GAE) per g essential oil. All determinations were carried out in triplicates and values were expressed as mean ± standard deviation (SD).

### Antioxidant activity (FRAP assay)

The antioxidant activity of essential oils was measured using the ferric reducing antioxidant power (FRAP) assay [28,29]. FRAP values of essential oil samples were performed using the extracts obtained previously for assessing of total phenolic compounds. Ferric to ferrous ion reduction at low pH (3.6 in acetate buffer) produces a colored ferrous-tripyridyltriazine complex. FRAP values are obtained by reading the absorbance changes at 595 nm which are linear over a wide concentration range. FRAP values were expressed as μM Fe^2+^ equivalents/g essential oil. All determinations were carried out in triplicates and values were expressed as mean ± standard deviation (SD).

### Statistical analysis

Data were reported as mean ± standard deviation. All analyses were performed in triplicate for each level and type of essential oil. Analysis of variance (ANOVA one-way) and the least significant difference test, in order to compare the mean values of the investigated parameters was carried out to find significant differences between fungus growth on wheat seeds after 5 and after 22 days of treatment. Computations Tukey post-hoc means comparisons and Levene’s test for equal variance was also included. Statistical processing data was performed using the Statistical Analysis System-SAS (Software version 8.1; SAS Institute, Inc.: Cary, NC, USA, 2000) [30]. Simple linear regression analysis performed by Origin 6.0 software was used for establishing of some correlations between investigated parameters.

## Results

### Impact of essential oils on natural mycoflora

Data concerning the effects of essential oils on natural mycoflora are presented in Table 1 and express the variation of SCI (%), registered relative to time and applied treatments. Activity of the each level of investigated essential oils was considered fungicidal if the pathogen did not grow, or fungistatic if the pathogen growth occurred [31].

**Table 1:** The changes of SCI (%) during storage as effect of treatment with essential oils

**Sample**		**SCI (%)**		
		**Period (days)**		
		**0**	**5**	**22**
Control		96,67 ± 5,77	96,67 ± 5,77^ns^	100,00 ± 0.00^ns^
O1	500 ppm	96,67 ± 5,77	43,33 ± 5,77***	100,00 ± 0,00^ns^
	1000 ppm	96,67 ± 5,77	20,00 ± 0,00***	100,00 ± 0,00^ns^
	2000 ppm	96.67 ± 5.77	0 ± 0.00^***^	56.67 ± 5.77***
O2	500 ppm	96,67 ± 5,77	36,67 ± 5,77^***^	100,00 ± 0,00 ^ns^
	1000 ppm	96,67 ± 5,77	26,67 ± 5,77^***^	86,67 ± 5,77^ns^
	2000 ppm	96.67 ± 5.77	10 ± 0.00***	33.33 ± 5.77***
O3	500 ppm	96,67 ± 5,77	33,33 ± 5,77^***^	100,00 ± 0.00^ns^
	1000 ppm	96,67 ± 5,77	30,00 ± 0,00^***^	100,00 ± 0,00^ns^
	2000 ppm	96.67 ± 5.77	20.00 ± 10.00***	63.33 ± 11.55*
O4	500 ppm	96,67 ± 5,77	36,67 ± 5,77^***^	100,00 ± 0,00^ns^
	1000 ppm	96,67 ± 5,77	33,33 ± 5,77^***^	100,00 ± 0,00^ns^
	2000 ppm	96.67 ± 5.77	20.00 ± 0.00***	93.33 ± 5.77^ns^
O5	500 ppm	96,67 ± 5,77	33,33 ± 5,77^***^	100,00 ± 0,00^ns^
	1000 ppm	96,67 ± 5,77	30,00 ± 0,00^***^	100,00 ± 0,00^ns^
	2000 ppm	96.67 ± 5.77	26.67 ± 5.77***	83.33 ± 5.77^ns^
O6	500 ppm	96,67 ± 5,77	36,67 ± 5,77^***^	66,67 ± 5,77^**^
	1000 ppm	96,67 ± 5,77	33,33 ± 5,77^***^	100,00 ± 0,00^ns^
	2000 ppm	96.67 ± 5.77	0.00 ± 0.00***	56.67 ± 5.77***

Statistical differences are indicated as: ns = non-significant (P > 0.1), * = significant (P < 0.05), ** = highly significant (P < 0.01) and *** = extremely significant (P < 0.001).

Examination of samples indicates the important role of essential oils at different concentrations in inhibiting the growth of fungi that are involved in the postharvest spoilage of wheat seeds. Generally, the inhibitory effect exhibited different intensity. Thus, after 5 days of treatment, there was recorded a decreasing in the SCI values both for filamentous fungi and yeasts. The treatments with essential oils to levels of 500, 1000 and 2000 ppm resulted in inhibition of fungal growth relative to control, the inhibition level was dependent on the essential oil type. This finding is in agreement to the results reported by Bluma *et al.*[20].

The highest inhibitory effect was registered for treatment with O1: SCI was 20.0% for a level of 1000 ppm and 0% for a level of 2000 ppm, relative to the value recorded for control sample (96.67%). The highest inhibition of fungal growth was noticed 5 days after treatment, when the differences was extremely significant (P < 0.001) related to the control. Lemon balm contains as principal component geraniol which showed previously antifungal effects [32].

The predominant compound of O2 is eucalyptol [33]. Besides this one, borneol, α-pinene and camphor were identified in O2 [34]. At 5 days after treatment with O2, fungal inhibition expressed by SCI was 10% for a level of 2000 ppm while this value became 26.67% for a level of 1000 ppm and 36.67% by using of this essential oil to a level of 500 ppm.

Monoterpenes are the main chemical compounds identified in O3. The essential oil obtained from mature fruits, at the final stage of maturity consists mainly in linalool (69.8-87.54%) which can be developed as a potential fumigant for stored-products protection [35,36]. By applying of O3 to all levels tested in this study, it was recorded an extremely significant inhibitory effect on fungal growth related to the control (P < 0.001). At 5 days after treatment with O3, SCI values were 33.33% for a level of 500 ppm, 30% for a level of 1000 ppm and 20% for a level of 2000 ppm.

Our results showed that after 5 days of treatment with O4 it was inhibited the growth of moulds in wheat samples. Thus, SCI values were in the range 36.67-20% depending on the applied level. Similar results on the antifungal effect of O4 have been reported by Dambolena *et al.*[37] and Kumar *et al.*[38]. However, statistical differences registered after 22 days of treatment are non-significant (P > 0.1) related to the control. Previous studies reported that phenolic compounds as thymol are responsible for the antifungal activity. Also, it contains a range of additional compounds, such as camphene, camphor, p-Cymene, myrcene, borneol and linalool [39,40]. The mechanism action of phenolic compounds includes enzyme inhibition by the oxidized compounds which affect the integrity of membrane, pH homeostasis and equilibrium of inorganic ions [41].

After 5 days of treatment with O5 it was possible to point out that SCI values decreased simultaneous with increasing of essential oil level (33.33% at a level of 500 ppm, 30% at a level of 1000 ppm and 26.67% at a level of 2000 ppm). The statistical differences recorded were extremely significant relative to the control. O5 contains higher amounts of menthol and eucalyptol [42]. The effect of menthol on the growth, sporulation and FUMO production has been previously proven [41].

In the case of treatment with O6, after 5 days of incubation the total populations of fungi were reduced. Thus, the values recorded for SCI were 36.67% for a level of 500 ppm and 33.33% by using of this essential oil to a level of 1000 ppm. The antifungal activity of O6 reached the maximum value for a level of 2000 ppm, proven by the value recorded for SCI (0%). According to results obtained after statistical processing of data by one-way ANOVA test, the differences related to the control were extremely significant. The exhibited antifungal activity was consistent with other studies which has highlighted that O6 to a level at least 1000 ppm induced a total inhibition of the fungus growth. In agreement with data reported by Soliman and Badeaa [17], we could attribute this effect to the main chemical components such as eugenol, eugenol acetate, cinnamic aldehyde and benzyl benzoate identified in this essential oil.

We can notice that after 22 days of treatment the inhibitory effect induced by applying of all essential oils on fungal contamination decreased. This finding can be explained by evaporation of the active principles specified to essential oils. The statistical differences recorded in the natural mycoflora of wheat grains treated with essentials oils were non-significant (P > 0.1) relative to the control. Only for the level of 2000 ppm were recorded significant differences for O3 and extremely significant for O1, O2 and O6.

### Impact of essential oils on frequency of fungus genera

**Figure 1:**
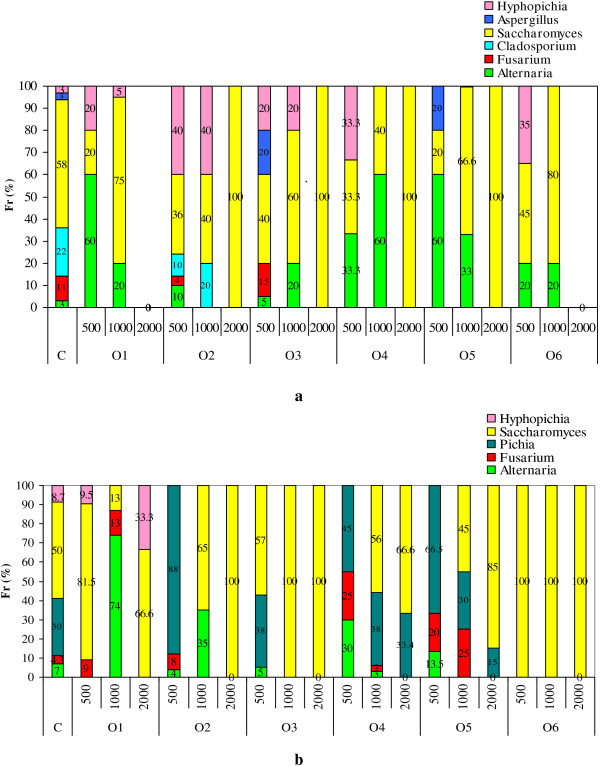
Frequency of fungus genera (a: 5 days after treatment; b: 22 days after treatment).

The screening of natural mycoflora developed on the wheat seeds allowed to estimate the relative frequency, Fr (%), of fungal genera after 5 and 22 days of incubation. The obtained data are presented in Figure 1. These results lead to the assumption that each treatment with tested essential oils produces a selective inhibitory effect on fungi developed on wheat seeds. Thus, after 5 days, the most identified genera in the control sample, in terms of frequency, were *Saccharomyces* (58%) followed by *Cladosporium* (22%). Other genera such as *Fusarium* (11%) *Aspergillus* (3%), *Alternaria* (3%), *Hyphopichia* (3%) were represented by frequency values smaller than two genera reported previously.

To a closer inspection of obtained data we can notice that O1 inhibited the growth of both *Fusarium* and *Aspergillus* species for all levels used in vivo treatments. Recent studies carried out in vitro pointed out that the treatment with lemon balm essential oil not resulted in a fungicidal effect for a concentration of 7.5 mg∙ml^-1^[43].

In the case of treatment with O1 to a level of 500 ppm, *Alternaria* was the major fungus present in wheat samples, while for a level of 1000 ppm the yeast *Saccharomyces* was the most predominant. The treatment with this essential oil to a level of 2000 ppm has inhibited completely the germination of spores and the growth of molds.

The treatment with O2 to a level over 500 ppm inhibited the growth of *Fusarium, Alternaria*, and *Asppergillus* comparatively with O4, O5 and O6. Similar results were reported by Daferera *et al.*[34] regarding the effects of sage essential oils on mycelial growth of *Fusarium sp*. Thus, it was noticed that the treatment with O2 to a level over 1000 μg·ml^-1^ induced a decreasing of 50% of mycelium linear growth of *Fusarium.* Based on our data it be seen that by applying of O2 to a level of 2000 ppm, *Saccharomyces* species are the most tolerant fungus. This result strengthens the finding reported by Vukovic *et al.*[44] on the fact that treatment with sage extract exhibited a protective effect on *Saccharomyces cerevisiae.*

The treatment with O3 applied for wheat seed samples favored a high frequency of occurrence of fungi such as *Alternaria*, *Fusarium, Saccharomyces*, *Aspergillus* and *Hyphopichia*. Only to a level of 2000 ppm it was noticed a decreasing of the fungus frequency. According to other data reported by Zoubiri *et al.*[36] we have supposed that this effect was due to linalool - the main compound identified in O3.

The O4 exhibited a broad spectrum fungitoxicity against different fungi. After 5 days of treatment no *Fusarium, Cladosporium* and *Aspergillus* species were detected. The results were similar with those reported by other previous researches [1,38], that indicated a complete inhibition of the growth of *Aspergillus flavus*, *Fusarium osysporum*, *Curvularia lunata, Aspergillus terreus, Aspergillus fumigantus, Alternaria* and *Cladosporium* sp*.* by treatment with different concentrations of O4. Also, the growth and spore germination of *Aspergillus niger, A. ochraceus* and *A. flavus* were fully inhibited by the thyme oil to a level of 600 ppm [20]. For treatments carried out in *vitro*, the fungitoxicity of O4 was recorded for a level of 71 μg∙ml^-1^. For this level it was recorded a decreasing of 50% of linear growth of *Fusarium* species. Considering this aspect, many researchers recommend the treatment with O4 as a natural way to control the presence of micotoxingenic fungi in stored cereals [34,38].

Menthol is a naturally occurring compound present in the volatile oil of several species of mint such as *Mentha piperita*. After 5 days of treatment, the species identified in the sample treated with O5 to a level of 500 ppm belong to genera: *Alternaria, Aspergillus* and *Saccharomyces.* In samples treated with O5 to levels of 1000 and 2000 ppm were predominant only *Alternaria* and *Saccharomyces* genera. Other previous studies reported that menthol stereoisomers and menthone exhibited no significant antitoxigenic activity that may be related to their structural type and the functional of present group [40,41]. In order to prevent the moulds growth is needed the doses of essential oils higher than 1000 ppm.

The treatment with O6 inhibited the growth of *Fusarium* and *Cladosporium* species depending on applied level. It was noticed that *Alternaria* species were more resistant to treatment with O6 than others filamentous fungus identified on the wheat seeds treated with this essential oil to a level of 500 and 1000 ppm. The highest level of O6 (2000 ppm) inhibited both yeasts and moulds. Our results are in agreement with those of previous studies that reported antifusarium activity against non-toxigenic (*F. solani* and *F. oxysporum*) and toxigenic (*F. verticillioides*, *F. poae* and *F. culmorum*) isolates [14]. Also, the antifungal activity against *Cladosporium herbarum, Rhizopus* and *Aspergillus niger* was reported [45].

The treatments with O5 and O6 to a level of 500 ppm resulted in the identification of the *Aspergillus sp*. with a frequency of genera about 20%. The most fungicidal effect on *Aspergillus* growth was recorder for treatment with O4, followed by O1 and O6 while O5 and O3 were able to induce an inhibition just over the level of 500 ppm.

After 22 days of incubation in dark conditions, at 25 ± 2°C and water activity (a_w_) to a value of 0.900 the frequency of occurrence of the fungus genera on wheat grain samples has changed in terms of fungal colonization. In control sample were identified filamentous fungi belonging to the *Alternaria si Fusarium* genera and fungus such as yeasts *Pichia, Saccharomyces* and *Hyphopichia*. Overall, it has been observed a great abundance of *Saccharomyces* for all concentrations of essential oils. In case of treatments with essential oils to a level of 2000 ppm it was noted that the growth of *Alternaria* and *Fusarium* was fully inhibited while *Hyphopichia* and *Saccharomyces* genera showed a high tolerance. It seems that to a level of 2000 ppm all essential oils induced a strong antifungal effect because there it was not observed the fungal growth of filamentous fungus. The antifungal effect of these essential oils on yeast *Pichia, Saccharomyces* and *Hyphopichia* has been reached to a level of 2000 ppm while the treatments with lower doses of essential oils induced a fungistatic effect.

The studies conducted in vitro found that the fungicidal effect expresses as minimum inhibitory concentration started from a level of 500 ppm for cinnamon oil [17]. Thus, in order to reach the fungicidal effect in the case of in vivo treatments is needed the higher levels of essential oils than for in vitro applications.

In case of thyme essential oil applied in vitro treatments, the fungicidal effect of *Fusarium moniliforme* was reached at a level of 125 ppm [17]. In our study, carried out in vivo, it was needed the doses higher than 500 ppm in order to inhibit the growth of *Fusarium* species, as can be seen in Figure 1a. For treatment with lemon balm essential oil the antifungal effect was reached to a level over 1000 ppm, and for peppermint essential oil the same effect was noted to a level of 500 ppm. Also, it was demonstrated that cinnamon essential oil had a significant inhibitory effect on growth of *F. proliferatum* at a_w_ of 0.995. The inhibitory effect was significant at levels of 500 and 1000 ppm [46].

The antifungal effect of essential oils could be explain by the modifications induced on the fungal morphogenesis and fungus growth through the interference of their components with the enzymes responsible for wall cell synthesis leading to changes in the hyphae integrity, plasma membrane disruption and mitochondrial destruction [1]. Antifungal activity of essential oils according to their major components was as follows: phenols > alcohols > aldehydes > ketones > ethers > hydrocarbons [32].

### Impact of essential oils on FUMO and DON production

**Figure 2:**
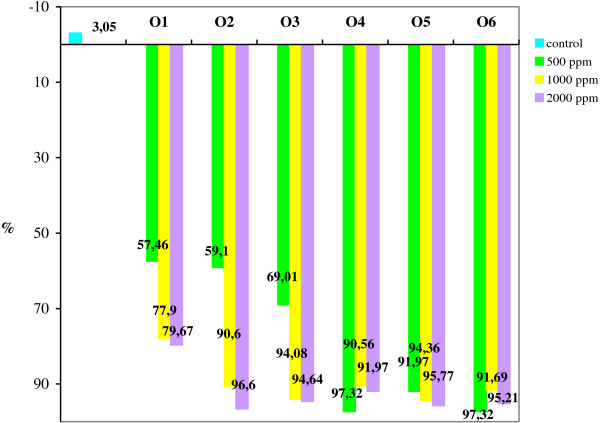
The decline registered in FUMO content by treatment with essential oils.

**Figure 3:**
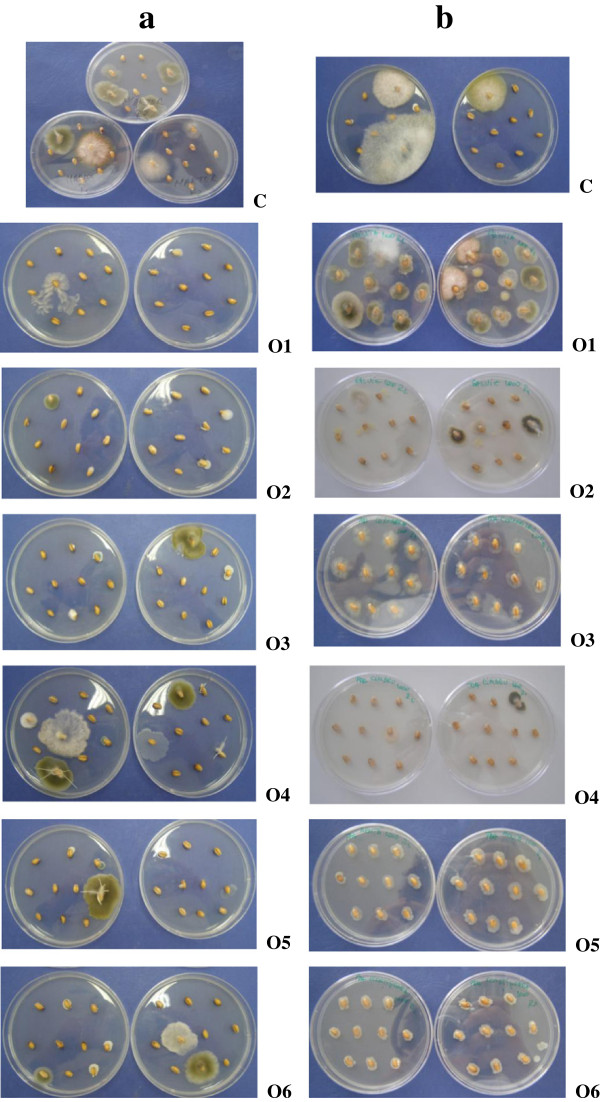
Fungal growth recorded for wheat samples treated with essential oils to a level of 1000 ppm (a: 5 days after treatment; b: 22 days after treatment).

The results proved that the treatment with essential oils resulted in decreasing of *Fusarium* mycotoxin in wheat seeds. Figure 2 provides information on the FUMO decline registered in response to treatment with essential oils relative to the control sample after 22 days of treatment. Fungal growth recorded for wheat samples treated with essential oils to a level of 1000 ppm is shown in Figure 3. At the beginning of the experiment the wheat seed samples were natural contaminated with 0.689 ppm FUMO and 0.420 ppm DON. After 22 days of treatment, the mycotoxin amount increased in the control sample up to 0.710 ppm for FUMO and recorded a small decreasing up to 0.416 ppm for DON.

The decline registered in FUMO content after 22 days of treatment with essential oils were in the range 57.46-97.32% related to the initial value depending on the type and level of the essential oil applied. The best control on FUMO production, expressed by a reduction over 90% related to the control, was recorded for all levels of O6, O5, and O4. These results are in agreement with the study of Soliman *et al.*[17] which found that the effect of essential oils in FUMO control was as follows: O4 > O6 > O5. The treatments with O1, O2 and O3 resulted in a moderate inhibitory effect for the lowest level of essential oils used in this study (500 ppm). Also, several researches have reported the preservation of grains by using of essential oils [17] and their impact on FUMO production by *F. verticillioides*[41,46,47] or by *F. proliferatum*[48].

Our results shown that the losses of FUMO recorded in wheat samples as a result of treatment with O1 were in the range 57.46-79.67% related to the initial value. Similar results were also noticed for treatments with O2 and O3 at a level of 500 ppm, while the inhibitory potential to the doses of 1000 and 2000 ppm was maintained in the range 90.6-96.6%.

The mycotoxins production is affected by the treatment conditions (temperature and moisture content of the grains). It might be assumed that the penetration of the oils into the internal parts of the grain is improved in the presence of water. Previous researches proved that a_w_, temperature, dose and type of essential oil as well as some of their interactions had a significant effect on FUMO production by *F. proliferatum*[46,49]. In our study, the constant conditions, in terms of a_w_ (0.900) and temperature (25 ± 2°C), resulted in a decreasing of DON and FUMO content in wheat samples after 22 days.

As regards the effect of essential oil composition on mycotoxin synthesis, on the one hand a few studies have reported high inhibitory activity exhibited by phenolic compounds (cyclic terpenes, eugenol, carvacrol and their isomer thymol). The mechanism of phenolic compounds supposes the involvement of these compounds in enzyme inhibition, possibly through reaction with sulfhydryl groups or through interactions with proteins [37]. On the other hand, it has reported that the relative antifungal activity of the essential oils can not be easily correlated with any individual component but just with a mixture of compounds from these oils [50,51].

Inhibition of fungus growth and toxins production do not always occur together [12]. In our study, after 22 days of treatment, O4-O6 produced the higher FUMO inhibition, but the most fungicidal effect was recorded for O2 to a level of 2000 ppm. For example, previous studies with *F. culmorum* and *F. graminearum* pointed out that growth of fungi was significantly inhibited by cinnamon essential oil, but toxin production was enhanced [41]. Also, Magan *et al.*[21] found that suboptimal levels of fungicides stimulated DON production by *F. culmorum* in wheat grains. The additional stress of the fungicidal agents combined with water stress could stimulate mycotoxin production [11]. The inhibitory effect of terpenes on *Fusarium* growth and FUMO production followed the sequence: limonene > thymol > menthol > menthone [37]. According to our findings O4 contains high amounts of thymol, as noted previously [37] exhibited significant inhibitory effect on FUMO biosynthesis (expressed by a loss of 97.32% of the initial value to a level of 500 ppm).

After 22 days of treatment with essential oils, DON was undetectable in all wheat samples. Similar effect of essential oils on growth rate of DON produced by *Fusarium* species was reported [46]. The inhibition of DON production in control sample can be explained by the maintaining of a_w_ to a value of 0.900 during the entire period of incubation, other previous studies proving that the minimum value of a_w_ for DON production by *Fusarium sp* seems to be limited about 0.93 at 25°C [49].

### Antioxidants properties of essential oils

In Table 2 are presented the antioxidant activity expressed as ferric reducing antioxidant power (FRAP), as well as the total phenolic content (TP) for all essential oils used in this study. Due to their complex composition, the antioxidant properties of the essential oils cannot be evaluated only by one method [52]. Thus, TP and FRAP value were used for screening of antioxidant properties of essential oils tested in this paper. The inhibitory effect on fungus growth and mycotoxins production was associated with antioxidant properties of investigated essential oils. From our data it can be noticed that O4 exhibited the highest FRAP value (650.48 μM Fe^2+^ · g^-1^) followed by O1 (246.23 μM Fe^2+^ · g^-1^) and O6 (230.03 μM Fe^2+^ · g^-1^). The high antioxidant activity of these essential oils could be attributed to phenolic components (mainly, carvacrol and thymol) and their hydrogen donating ability by which they are considered powerful free radical scavengers [53,54]. O2 and O3 showed lower values recorded for FRAP than other investigated essential oils. Our findings are in agreement with the results reported by Hussain *et al.*[55], who noted that the antioxidant activity of essential oil from *Salvia officinalis* showed less radical scavenging activity than other *Lamiaceae* species. Contrary to other data reported by Chia-Wen *et al.*[53], in our study O1 exhibited a higher antioxidant activity. According to our results, the highest TP content was noticed for O4 *(*473.44 μM GAE · g^-1^) while the values recorded for other investigated essential oils were in the range 16.71-33.01 μM GAE · g^-1^. Many studies have reported variable phenolics content in essential oils as follows: O1 (19.93-499.64 μM GAE · g^-1^), O2 (142.84 μM GAE · g^-1^), O4 (67.83 μM GAE · g^-1^), O5 (13.40-526.69 μM GAE · g^-1^) and O6 (774.04 μM GAE · g^-1^) [54,55].

**Table 2:** Antioxidant characteristics of essential oils

**Essential oils**	**Total phenolics (μM GAE∙g**^**-1**^**)**	**Antioxidant activity (μM Fe**^**2+**^**∙g**^**-1**^**)**
O1	33.01 ± 2.52	246.23 ± 9.37
O2	18.52 ± 1.06	55.48 ± 3.81
O3	16.71 ± 0.93	40.41 ± 2.73
O4	473.44 ± 11.27	650.48 ± 14.29
O5	22.48 ± 1.63	100.85 ± 5.21
O6	30.17 ± 2.41	230.03 ± 8.12

According to data shown in Table 2, the antioxidant properties of essential oils were as follows: O4 > O1 > O6 > O5 > O2 > O3. Geographical area and culture conditions influence the chemical composition as well as and the antioxidant properties of medicinal plants [56]. For these reasons there are differences in the results obtained by different authors. Politeo *et al.* reported the following sequence in terms of antioxidant activity of essential oils: O4 > O5 > O6 > O2 [57].

**Table 3:** Correlation coefficients obtained by linear regression analysis applied for investigated parameters

**Correlation**	**Correlation coefficient (r)**					
	**5 days after treatment**					
Y = A + BX	**O1**	**O2**	**O3**	**O4**	**O5**	**O6**
FRAP = f(SCI)	−0,92	−0,64	−0,79	−0,82	−0,73	−0,91
TP = f(SCI)	−0,88	−0,87	−0,79	−0,80	−0,74	−0,91
	**22 days after treatment**					
Y = A + BX	**O1**	**O2**	**O3**	**O4**	**O5**	**O6**
FRAP = f(SCI)	−0,88	−0,99	−0,88	−0,88	−0,88	−0,63
TP = f(SCI)	−0,88	−0,94	−0,88	−0,89	−0,88	−0,63
FRAP = f(FUMO)	−0,78	−0,84	−0,81	−0,65	−0,69	−0,68
TP = f(FUMO)	−0,81	−0,84	−0,81	−0,65	−0,71	−0,68

### Correlations

Simple linear regression analysis was applied using the Origin 6.0 software program. Table 3 shows the values of linear correlation coefficients or Pearson's correlation coefficients (r) obtained as a response to linear regression between: FRAP and SCI recorded after 5 and 22 days of treatment, TP and SCI recorded after 5 and 22 days of treatment, FRAP and FUMO after 22 days of treatment and TP and FUMO registered after 22 days of treatment. The Pearson’s correlation coefficient (r) represents a quantitative measure to describe the strength of the linear relationship established between investigated parameters. By comparing of the values of these coefficients obtained in response to regressions established between investigated parameters recorded after 5 days of treatment, it was noticed that there was recorded a significant negative linear correlation between antioxidant properties expressed by FRAP or TP and SCI for O1 and O6 (r > 0.88), suggesting that essentials oils with high antioxidant characteristics induced a low fungal contamination.

Using O2-O5, the values recorded for correlation coefficients were in the range 0.64-0.87 representing a low to medium correlation between antioxidant properties of applied oils and SCI.

After 22 days, according to these values, resulted a significant correlation (r > 0.87) between antioxidant activity and the fungal load induced by using of most essentials oils, except cinnamon oil (O6).

Based on regression analysis between antioxidant properties of essential oils and FUMO content recorded in grain samples after 22 days it can be noticed that the correlation coefficients did not exceed the value of 0.844 for all essentials oils. This fact proves that the high antimycotoxin activity of the essential oil could be due to other components, major and minor, or by the synergistic effect of their which can act together for biological activity of essential oils, as suggested by Prakash *et al.*[58], Rota *et al.*[59] and Velluti *et al.*[46]. Although, the essential oils were not as efficiently as some organic preservatives, they are recommended in food technologies due to absence of toxic effect.

Regarding the correlation between *Fusarium* mycotoxins expressed by FUMO content and antioxidant activity of essential oils, there was not recorded a good correlation. This fact could suggest that TP and FRAP have not a crucial role in expression of antimycotoxin properties of these essential oils. Although it was noticed a high positive linear correlation between FRAP and TP of investigated essential oils (R = 0.94), besides polyphenolic compounds there are others responsible for their antioxidant properties that might be involved in the expression of inhibitory potential [57].

## Conclusions

Based on our data, this work could be an important tool for assessment of essential oils inhibitory potential on the fungal growth and *Fusarium* mycotoxins production in natural contaminated wheat. By applying the treatment with essential oils it was noticed that essential oils from cinnamon and lemon balm exhibited a significant antifungal activity. The highest inhibition of fungal growth was observed after 5 days of treatment and decrease after 22 days, probably due to high volatility of essential oils. Regarding the frequency of occurrence of fungus genera on wheat seeds, it was proven that O2 shown the highest inhibitory potential on the growth of *Fusarium*, *Alternaria*, and *Asppergillus* to a level of 500 ppm. In terms of the impact of essential oils on mycotoxin production, at the end of treatment it was recorded the inhibition of DON and FUMO production. The best control on FUMO production was noted in samples treated with O6 followed by those treated with O5 and O4. It was found that the essential oil having the best antifungal properties has not proved to be the most effective inhibitor in *Fusarium* mycotoxin production. It was noticed a significant negative linear correlation between antioxidant and seed contamination index for O1 and O6, suggesting that essentials oils with high antioxidant characteristics induced a low fungal contamination. Contrary, it was not recorded a good correlation between FRAP/TP and FUMO content suggesting that antioxidant properties of essential oils have not a crucial role in expression of antimycotoxin effect. As a result of this study, the essential oils may be recommended as natural preservatives applied during cereals storage.

## Abbreviations

O1: Essential oil from *Melissa officinalis* L.; O2: Essential oil from *Salvia officinalis* L*.*; O3: Essential oil from *Coriandrum sativum* L*.*; O4: Essential oil from *Thymus vulgaris* L.; O5: Essential oil from *Mentha piperita* L.; O6: Essential oil from *Cinnamomum zeylanicum* L*.*; SCI: Seed contamination index; Fr: Isolation frequency of genera; a_w_: Water activity; FUMO: Fumonisin; DON: Deoxynivalenol; FRAP: Ferric reducing antioxidant power; TP: Total phenolics; C: Control (untreated sample); PDA: Medium, potato dextrose agar; DCPA: Dichloran chloramphenicol peptone agar medium.

## Competing interests

The authors declare that they have no competing interests.

## Authors' contributions

RMS performed microbiological tests, fungus identification, helped to data processing and interpretation. EA performed mycotoxins analysis, contributed to oil analysis and results interpretation. MAP performed analysis on antioxidant characteristics of essential oils, statistical processing and contributed to data interpretation. All authors performed manuscript preparation, read and approved the final version of this one.
